# Three-year longitudinal assessment of denosumab’s efficacy and safety in postmenopausal osteoporosis: a 36-month real-world cohort study

**DOI:** 10.3389/fmed.2026.1825383

**Published:** 2026-07-14

**Authors:** Rong-Lin Xia, Chen Li, Li Wang, Ai-Jun Chao, Wei Wei

**Affiliations:** 1 Department of Rheumatology and Immunology, Tianjin Medical University General Hospital, Tianjin, China; 2 Department of Osteo-Internal Medicine, Tianjin Hospital, Tianjin, China; 3 Department of Orthopaedics, Tianjin Hospital, Tianjin, China

**Keywords:** 36-month therapy, adherence, bone mineral density, bone turnover markers, denosumab, osteoporosis, postmenopausal women, safety

## Abstract

**Background:**

Denosumab is an effective antiresorptive therapy for postmenopausal osteoporosis; however, long-term real-world data regarding sustained efficacy, treatment adherence, safety, and biochemical outcomes remain limited. This study evaluated the 36-month effectiveness and safety of denosumab in postmenopausal women with osteoporosis receiving routine clinical care.

**Methods:**

This retrospective analysis included a prospectively followed real-world clinical cohort of 130 postmenopausal women with osteoporosis treated with denosumab (60 mg subcutaneously every 6 months) plus calcium and vitamin D supplementation. Participants were followed for 36 months with assessments at baseline, 12, 24, and 36 months. Outcomes included bone mineral density (BMD), T-scores, bone turnover markers, renal function, adherence patterns, prior treatment history, fracture occurrence, and adverse events. Changes over time were analyzed using paired longitudinal comparisons and linear mixed-effects models, with significance defined as *p* < 0.05.

**Results:**

Lumbar spine (L1–L4) BMD increased from 0.674 ± 0.120 to 0.741 ± 0.102 g/cm^2^, while femoral neck BMD increased from 0.526 ± 0.086 to 0.562 ± 0.079 g/cm^2^ over 36 months (both *p* < 0.001). BMD improvements were accompanied by reductions in bone turnover markers, including *β*-CTX and PINP, indicating sustained suppression of bone resorption. No clinically meaningful renal deterioration was observed during follow-up. Twenty-one participants had prior osteoporosis treatment history, whereas all included patients completed seven denosumab injections. Delayed injections occurred in 29 patients, although most delays were <30 days. No new fragility fractures occurred during follow-up. No cases of osteonecrosis of the jaw, atypical femoral fracture, or injection-related serious adverse events were observed. Mild hypocalcemia occurred in several patients and resolved with monitoring and supplementation adjustment.

**Conclusion:**

Thirty-six months of denosumab treatment was associated with sustained improvement in BMD, suppression of bone turnover, acceptable adherence, and a favorable safety profile in postmenopausal women with osteoporosis who completed the planned 36-month treatment course. Because only patients completing all seven injections with complete follow-up data were included, adherence, persistence, and safety outcomes may be overestimated compared with an intention-to-treat population. These findings support the effectiveness of denosumab under routine clinical practice while emphasizing the importance of continued monitoring and adherence management.

## Graphical abstract

**Figure:**
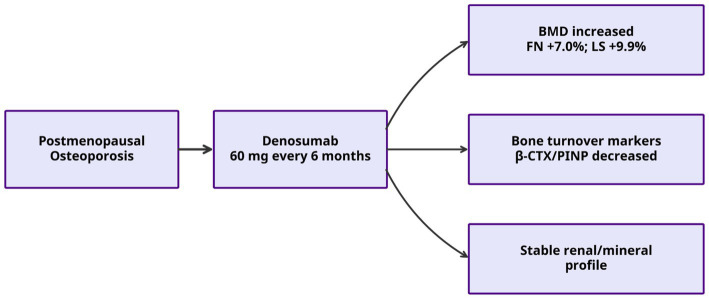

## 1 Introduction

Osteoporosis is a major global health concern characterized by reduced bone mass, deterioration of bone microarchitecture, and increased susceptibility to fragility fractures. It substantially reduces quality of life and increases morbidity and mortality, particularly among older adults (1, 2). Because bone loss is often clinically silent until fracture occurs, osteoporosis is frequently underdiagnosed and undertreated despite its high disease burden.

Globally, osteoporosis affects approximately one in three women and one in five men over the age of 50 (2). A comprehensive systematic review estimated the global prevalence of osteoporosis at 18.3%, with a higher prevalence in women than in men (3). With population ageing, the clinical and economic burden of osteoporotic fractures is expected to continue increasing, particularly among postmenopausal women and elderly populations (1–3).

Current management of postmenopausal osteoporosis integrates lifestyle modification, adequate calcium and vitamin D intake, fall prevention, fracture-risk assessment, and pharmacological therapy (4, 5). Antiresorptive therapy remains a cornerstone of treatment, particularly in patients at increased fracture risk. Denosumab is a fully human monoclonal antibody against receptor activator of nuclear factor κB ligand (RANKL), thereby inhibiting osteoclast formation, function, and survival and reducing bone resorption (6). It is administered as a 60 mg subcutaneous injection every 6 months and has been widely used for the treatment of postmenopausal osteoporosis.

The pivotal FREEDOM trial demonstrated that denosumab significantly reduced vertebral, non-vertebral, and hip fracture risk in postmenopausal women with osteoporosis (7). Long-term extension studies further showed progressive increases in bone mineral density (BMD) with continued denosumab therapy over prolonged follow-up (6, 8). Treat-to-target approaches, including achieving clinically meaningful BMD thresholds, have also been proposed to guide long-term osteoporosis management and fracture-risk reduction (9).

Despite strong evidence from randomized trials and extension studies, real-world longitudinal data remain important because routine clinical practice differs from controlled trial settings. Real-world patients may have prior exposure to anti-osteoporosis treatments, variable adherence to the 6-month injection schedule, differences in supplementation, and heterogeneous comorbidity profiles. In addition, long-term denosumab management requires careful monitoring because delayed dosing or discontinuation may lead to rebound bone turnover, rapid BMD loss, and increased vertebral fracture risk (10, 11).

Therefore, additional intermediate-term real-world evidence is needed to evaluate BMD trajectories, bone turnover suppression, adherence patterns, renal and mineral metabolism parameters, and safety outcomes during prolonged denosumab therapy. To address these issues, we conducted a retrospective analysis of a prospectively followed 36-month real-world clinical cohort of postmenopausal women with osteoporosis receiving denosumab in routine clinical care. This study assessed changes in femoral neck, lumbar spine, and total hip BMD; changes in T-scores and bone turnover markers; treatment adherence and injection delays; fracture occurrence; and renal, mineral, and adverse-event outcomes over 36 months.

## 2 Materials and methods

### 2.1 Study design and ethical framework

This study was designed as a retrospective analysis of a prospectively followed real-world clinical cohort of postmenopausal women with osteoporosis treated with denosumab at Tianjin Hospital, Tianjin, China. Patients received denosumab as part of routine clinical care between January 2020 and December 2023. The present analysis of anonymized longitudinal clinical data was approved by the Tianjin Hospital Medical Ethics Committee on 20 August 2025 (approval no. 2025 YLS111). The ethics committee confirmed that the study involved analysis of existing clinical data and caused no additional harm to participants.

A total of 134 postmenopausal women entered follow-up. Four patients were excluded from the final analysis because of incomplete longitudinal clinical or laboratory data. Therefore, 130 patients with complete evaluable 36-month data were included in the final analysis.

### 2.2 Participants

Eligible participants were postmenopausal women aged 49–85 years with osteoporosis, defined as a dual-energy X-ray absorptiometry (DXA)-derived T-score ≤ − 2.5 at the femoral neck or lumbar spine (L1–L4). Exclusion criteria included impaired renal function, defined as estimated glomerular filtration rate <35 mL/min/1.73 m^2^; hepatic dysfunction, defined as alanine aminotransferase or aspartate aminotransferase >3 times the upper limit of normal; metabolic bone diseases other than postmenopausal osteoporosis; recent use of bone-modifying agents; severe vitamin D deficiency; incomplete follow-up; or incomplete longitudinal clinical or laboratory data.

Previous anti-osteoporosis treatment history was recorded. Among the final analyzed cohort, 21 patients had previously received anti-osteoporosis therapy before denosumab initiation. Fourteen patients had previously received bisphosphonates for 5 months to 2 years, all of whom had discontinued treatment for more than 2 years before enrollment. Four patients had previously received teriparatide for 3–12 months and had discontinued treatment for more than 1 year. Three patients had previously received short-term menatetrenone/vitamin K2 therapy for 1 month to 1 year and had discontinued treatment for more than 1 year. The remaining patients were considered treatment-naïve for anti-osteoporosis pharmacotherapy at study entry.

### 2.3 Intervention and follow-up

All included patients received denosumab 60 mg by subcutaneous injection every 6 months for a total of seven injections over 36 months. Calcium and vitamin D supplementation were administered according to routine clinical practice. Treatment adherence was assessed using scheduled injection records and clinic verification. An injection administered within ±15 days of the scheduled date was considered on schedule, whereas administration beyond this window was defined as delayed.

Because the objective of this study was to evaluate intermediate-term denosumab outcomes in patients able to complete the planned 36-month treatment course, only patients who completed the planned treatment course and had complete evaluable follow-up data were included in the final analysis. This design feature was considered when interpreting persistence and safety outcomes.

### 2.4 Outcome measures

The co-primary endpoints were changes in femoral neck BMD and lumbar spine BMD from baseline to 36 months. Secondary endpoints included changes in total hip BMD, skeletal T-scores, bone turnover markers, renal and mineral metabolism parameters, treatment adherence, incident fractures, and safety events.

All DXA examinations were performed using the same densitometer by trained operators according to standardized institutional quality-control procedures throughout the study. Complete paired DXA measurements were available for 124 participants, whereas laboratory analyses included all available measurements from the full cohort of 130 participants.

BMD was measured by DXA at the femoral neck, lumbar spine (L1–L4), and total hip. Bone turnover markers included *β*-C-terminal telopeptide (β-CTX) and procollagen type I N-terminal propeptide (PINP). Additional biochemical parameters included parathyroid hormone, creatinine, urea, uric acid, serum calcium, phosphate, alkaline phosphatase, and 25-hydroxyvitamin D.

Prior fragility fracture was defined as a non-traumatic or low-trauma fracture occurring after menopause, excluding craniofacial fractures such as skull, jaw, or nasal bone fractures. Incident fractures during follow-up were classified as fragility fractures or traumatic fractures according to clinical history and mechanism of injury.

### 2.5 Safety monitoring

Safety outcomes included hypocalcemia, hypercalcemia, renal deterioration, osteonecrosis of the jaw, atypical femoral fracture, injection-site infection, serious adverse events, and incident fractures. Serum calcium and renal function were monitored during routine follow-up assessments. Calcium abnormalities were recorded according to laboratory values and clinical review. Cases of abnormal calcium related to excessive supplementation were managed by adjustment or discontinuation of supplementation, followed by reassessment. Serum calcium was assessed during routine clinical follow-up after denosumab administration; however, systematic early post-injection calcium testing within 2–4 weeks was not performed for all patients.

### 2.6 Statistical analysis

Continuous variables were summarized as mean ± standard deviation (SD), and categorical variables were summarized as number and percentage. Baseline-to-36-month paired comparisons were performed for patients with available paired measurements at both timepoints, and mean differences were reported with 95% confidence intervals (CIs). Because the study involved repeated longitudinal measurements within the same patients, linear mixed-effects models were additionally used to evaluate time-dependent changes across follow-up. In these models, time was included as a fixed effect and patient identity as a random intercept to account for within-subject correlation. The final analyzable cohort of 130 patients was considered adequate for evaluating longitudinal within-patient changes in BMD; however, the study was not powered to assess fracture endpoints because of the relatively small cohort size and the low expected incidence of fractures during the 36-month follow-up period. Results are presented as estimated annual change, 95% CI, and *p* value. All tests were two-sided, and *p* < 0.05 was considered statistically significant.

## 3 Results

### 3.1 Patient characteristics and study flow

A total of 134 postmenopausal women with osteoporosis entered follow-up. Four patients were excluded because of incomplete longitudinal clinical or laboratory data, resulting in a final analyzed cohort of 130 patients. All included patients completed seven scheduled denosumab injections and the 36-month follow-up assessment.

Mean baseline height was 159.2 ± 4.4 cm. Twenty-one patients had a history of prior anti-osteoporosis therapy before denosumab initiation, including 14 previously treated with bisphosphonates, four with teriparatide, and three with menatetrenone/vitamin K2. All previous therapies had been discontinued for at least 1–2 years before enrollment, depending on treatment type. Twenty-seven patients had a documented history of prior fragility fracture at baseline. Baseline cohort characteristics, prior treatment exposure, adherence, fracture outcomes, and safety events are summarized in Table 1.

**Table 1:** Clinical cohort characteristics, prior treatment, adherence, fractures, and safety outcomes.

Variable	Result
Patients entering follow-up	134
Excluded due to incomplete data	4
Final analyzed cohort	130
Completed 36-month follow-up	130
Completed seven denosumab injections	130
Prior anti-osteoporosis treatment	21
Prior bisphosphonate therapy	14
Prior teriparatide therapy	4
Prior menatetrenone/vitamin K2 therapy	3
Prior fragility fracture history	27
Incident fragility fracture during follow-up	0
Traumatic fracture during follow-up	1
Any delayed injection	29
Delay of 15–30 days	22
Delay >30 days	7
Osteonecrosis of the jaw	0
Atypical femoral fracture	0
Injection-site infection	0
Hypocalcemia	9 patients
Hypercalcemia	3 patients

### 3.2 Treatment adherence and persistence

All patients included in the final analysis completed the planned seven denosumab injections. Using the predefined ±15-day window as the criterion for on-schedule treatment, 29 patients experienced at least one delayed injection during follow-up. Among these patients, 22 completed the delayed injection within 15–30 days, whereas seven had one delay exceeding 30 days. Eight patients had two delayed injections, all completed within 30 days. Two patients had three delayed injections; one completed all delayed injections within 30 days, whereas the other had a third delay of 41 days, with the remaining two delayed injections completed within 30 days. No patient discontinued denosumab during the analyzed follow-up period (Table 1).

### 3.3 Bone mineral density outcomes

After cleaning and verification of the longitudinal dataset, femoral neck BMD increased from 0.526 ± 0.086 g/cm^2^ at baseline to 0.562 ± 0.079 g/cm^2^ at 36 months among patients with paired measurements (*n* = 124). This corresponded to a mean increase of 0.037 g/cm^2^ (95% CI: 0.028 to 0.046; *p* < 0.001), or approximately 7.0%.

Lumbar spine BMD increased from 0.674 ± 0.120 g/cm^2^ to 0.741 ± 0.102 g/cm^2^ (*n* = 124), with a mean increase of 0.067 g/cm^2^ (95% CI: 0.055 to 0.078; *p* < 0.001), corresponding to an approximate 9.9% improvement. Total hip BMD also increased significantly from 0.670 ± 0.123 g/cm^2^ to 0.696 ± 0.109 g/cm^2^, with a mean increase of 0.026 g/cm^2^ (95% CI: 0.015 to 0.036; *p* < 0.001). Baseline-to-36-month skeletal and biochemical changes are presented in Table 2. Although total hip BMD increased significantly, the corresponding total hip T-score change did not reach statistical significance. Because T-scores are standardized values derived from reference population data and are primarily intended for diagnostic classification, small absolute BMD gains may not necessarily translate into statistically significant changes in T-score.

**Table 2:** Baseline-to-36-month changes in skeletal and biochemical outcomes.

Variable	Paired *n*	Baseline	36 months	Mean change (95% CI)	*p* value
Femoral neck BMD, g/cm^2^	124	0.526 ± 0.086	0.562 ± 0.079	0.037 (0.028 to 0.046)	<0.001
Lumbar spine BMD, g/cm^2^	124	0.674 ± 0.120	0.741 ± 0.102	0.067 (0.055 to 0.078)	<0.001
Total hip BMD, g/cm^2^	124	0.670 ± 0.123	0.696 ± 0.109	0.026 (0.015 to 0.036)	<0.001
Femoral neck T-score	124	−2.89 ± 0.71	−2.59 ± 0.69	0.30 (0.24 to 0.36)	<0.001
Lumbar spine T-score	124	−3.29 ± 0.99	−2.72 ± 0.97	0.58 (0.48 to 0.67)	<0.001
Total hip T-score	124	−2.13 ± 1.15	−2.02 ± 0.87	0.11 (−0.05 to 0.26)	0.187
β-CTX, ng/mL	130	0.631 ± 0.583	0.246 ± 0.235	−0.385 (−0.481 to −0.289)	<0.001
PINP, ng/mL	130	44.76 ± 32.79	20.41 ± 10.17	−24.35 (−29.72 to −18.98)	<0.001
Creatinine, μmol/L	130	60.16 ± 13.20	61.76 ± 11.19	1.61 (−0.06 to 3.28)	0.059
Calcium, mmol/L	130	2.46 ± 0.13	2.43 ± 0.16	−0.03 (−0.06 to 0.01)	0.099
Phosphate, mmol/L	130	1.17 ± 0.21	1.17 ± 0.20	−0.00 (−0.05 to 0.05)	0.923
25(OH)D, ng/mL	130	19.76 ± 8.68	38.36 ± 6.35	18.60 (16.73 to 20.47)	<0.001

Reference ranges: calcium, 2.10–2.55 mmol/L; phosphate, 0.81–1.45 mmol/L; creatinine, 44–80 μmol/L for women; β-CTX, 0.10–0.70 ng/mL; PINP, 15–60 ng/mL. Reference ranges may vary according to age, menopausal status, assay platform, and institutional laboratory standards.

Consistent improvements were also observed in skeletal T-scores. Femoral neck T-score improved from −2.89 ± 0.71 to −2.59 ± 0.69 (mean change: 0.30; 95% CI: 0.24 to 0.36; *p* < 0.001), and lumbar spine T-score improved from −3.29 ± 0.99 to −2.72 ± 0.97 (mean change: 0.58; 95% CI: 0.48 to 0.67; *p* < 0.001). Total hip T-score showed a smaller, non-significant change (mean change: 0.11; 95% CI: −0.05 to 0.26; *p* = 0.187) (Table 2).

### 3.4 Bone turnover markers

Denosumab treatment was associated with marked suppression of bone turnover markers over 36 months. Serum *β*-CTX decreased from 0.631 ± 0.583 ng/mL at baseline to 0.246 ± 0.235 ng/mL at 36 months, representing a mean reduction of −0.385 ng/mL (95% CI: −0.481 to −0.289; *p* < 0.001). PINP decreased from 44.76 ± 32.79 ng/mL to 20.41 ± 10.17 ng/mL, with a mean reduction of −24.35 ng/mL (95% CI: −29.72 to −18.98; *p* < 0.001). These findings indicate sustained suppression of bone remodeling activity during the 36-month treatment period (Table 2).

### 3.5 Renal and mineral metabolism outcomes

Renal function remained within clinically acceptable ranges throughout follow-up. Although the mixed-effects model identified a small statistically significant longitudinal increase in serum creatinine, the magnitude of change was minimal and no clinically meaningful deterioration in renal function was observed.

Serum calcium showed no significant overall decrease, changing from 2.46 ± 0.13 mmol/L to 2.43 ± 0.16 mmol/L (mean change: −0.03 mmol/L; 95% CI: −0.06 to 0.01; *p* = 0.099). Serum phosphate remained stable (mean change: −0.00 mmol/L; 95% CI: −0.05 to 0.05; *p* = 0.923). Serum 25(OH)D increased from 19.76 ± 8.68 ng/mL to 38.36 ± 6.35 ng/mL (mean change: 18.60 ng/mL; 95% CI: 16.73 to 20.47; *p* < 0.001), consistent with vitamin D supplementation (Table 2).

### 3.6 Linear mixed-effects model analysis

Linear mixed-effects models were used to evaluate longitudinal changes while accounting for repeated measurements within individual patients. Time was independently associated with increases in femoral neck BMD, lumbar spine BMD, and total hip BMD. Time was also significantly associated with reductions in *β*-CTX and PINP and with an increase in 25(OH)D. Serum calcium showed no significant longitudinal decline. Linear mixed-effects model results are presented in Table 3.

**Table 3:** Linear mixed-effects model of longitudinal changes over 36 months.

Variable	Observations	Subjects	Estimated annual change	95% CI	*p* value
Femoral neck BMD, g/cm^2^/year	518	130	0.0109	0.0085 to 0.0133	<0.001
Lumbar spine BMD, g/cm^2^/year	517	130	0.0223	0.0191 to 0.0255	<0.001
Total hip BMD, g/cm^2^/year	515	130	0.0088	0.0021 to 0.0155	0.010
β-CTX, ng/mL/year	910	130	−0.0940	−0.1086 to −0.0794	<0.001
PINP, ng/mL/year	910	130	−6.57	−7.46 to −5.68	<0.001
Creatinine, μmol/L/year	909	130	0.624	0.274 to 0.973	<0.001
Calcium, mmol/L/year	910	130	−0.0056	−0.0122 to 0.0010	0.094
25(OH)D, ng/mL/year	910	130	4.89	4.44 to 5.34	<0.001

Linear mixed-effects models included time as a fixed effect and patient identity as a random intercept.

### 3.7 Correlation analysis

**Figure 1:**
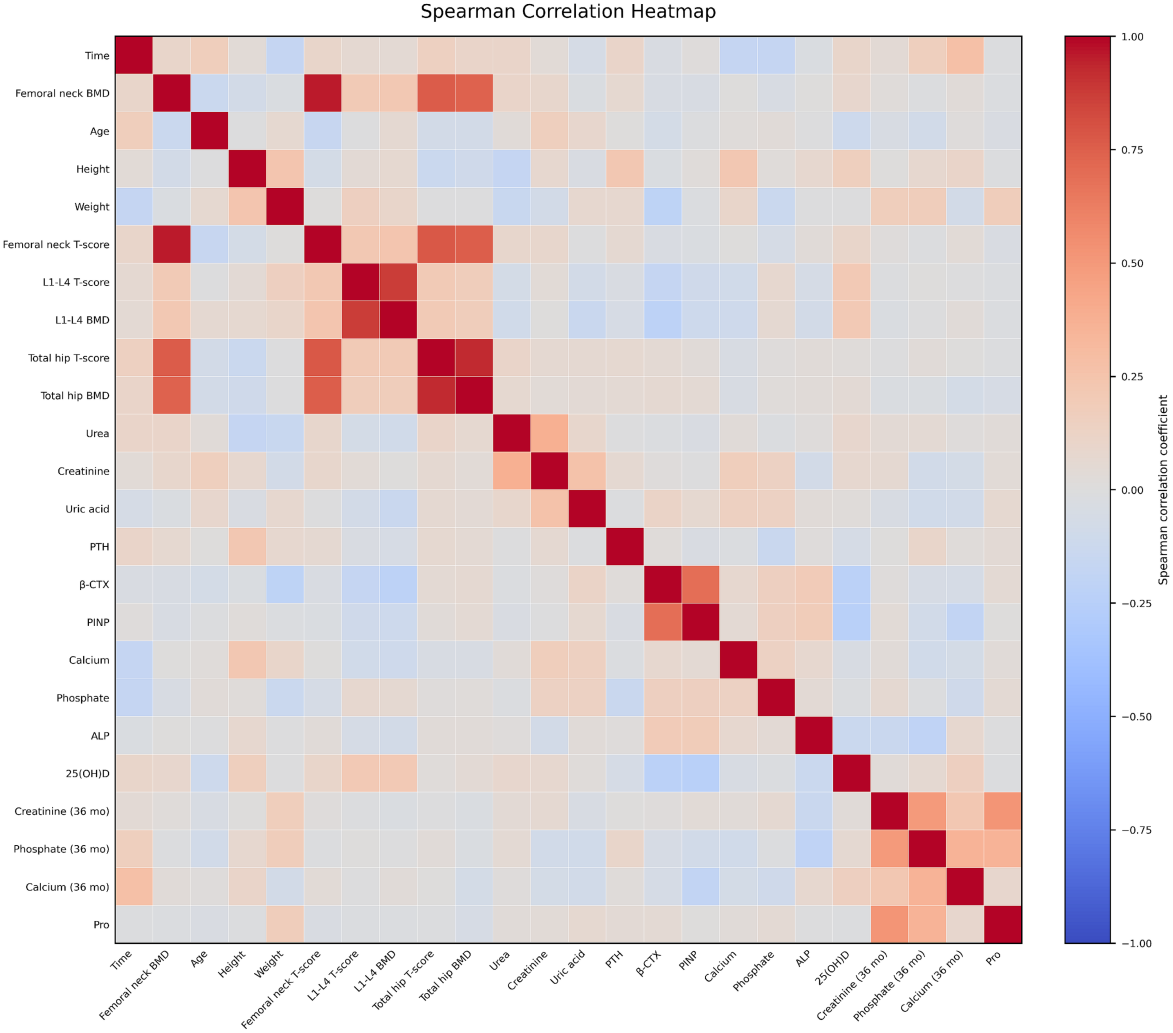
Spearman correlation heatmap illustrating relationships among key skeletal, bone turnover, renal, and mineral metabolism parameters during denosumab therapy. Warmer colors indicate positive correlations and cooler colors indicate negative correlations.

Spearman correlation analysis demonstrated strong positive correlations between femoral neck BMD and femoral neck T-score, lumbar spine BMD and lumbar spine T-score, and total hip BMD and total hip T-score. Bone turnover markers showed weak inverse correlations with BMD-related parameters. Correlation patterns among BMD, T-scores, bone turnover markers, renal markers, and mineral metabolism parameters are illustrated in Figure 1. The strongest positive correlations were observed between BMD measurements and their corresponding T-scores, whereas *β*-CTX and PINP demonstrated moderate positive intercorrelation and generally weak inverse associations with skeletal parameters.

### 3.8 Fracture and safety outcomes

During the 36-month follow-up, no incident osteoporotic fragility fractures were recorded. One patient sustained a traumatic tibia/fibula fracture caused by an electric-bike traffic accident; this event was not classified as an osteoporotic fragility fracture.

No cases of osteonecrosis of the jaw, atypical femoral fracture, or injection-site infection occurred during follow-up. Mild hypocalcemia was observed in nine different patients during routine post-injection follow-up. Two patients had low calcium after the second denosumab injection, with values of 2.12 and 2.06 mmol/L. One patient had low calcium after the third injection (2.13 mmol/L), one after the fifth injection (2.15 mmol/L), two after the sixth injection (2.15 and 2.16 mmol/L), and three after the seventh injection (2.11, 2.17, and 2.08 mmol/L). No severe symptomatic hypocalcemia was reported.

Three patients developed hypercalcemia during follow-up. Two cases, with calcium values of 2.96 and 2.83 mmol/L, were considered related to additional calcium carbonate intake of approximately 1,200 mg/day beyond diet. One case, with calcium of 3.04 mmol/L, was associated with self-administered vitamin D3 intake of approximately 5,000 IU/day. Calcium levels normalized after supplement adjustment or discontinuation. Fracture and safety outcomes are summarized in Table 1.

## 4 Discussion

### 4.1 Principal findings

In this retrospective analysis of a prospectively followed 36-month real-world cohort of postmenopausal women with osteoporosis receiving denosumab in routine clinical practice, treatment was associated with significant improvements in lumbar spine, femoral neck, and total hip BMD, accompanied by suppression of bone turnover markers. These findings are consistent with the established antiresorptive mechanism of denosumab, which inhibits receptor activator of nuclear factor κB ligand (RANKL), suppresses osteoclast formation and activity, and reduces bone resorption (6, 7). No clinically meaningful deterioration in renal function or mineral metabolism was observed, and no cases of osteonecrosis of the jaw, atypical femoral fracture, or injection-site infection were observed. Together, these findings support the intermediate-term effectiveness and tolerability of denosumab in a real-world postmenopausal osteoporosis cohort.

### 4.2 Interpretation in relation to previous evidence

The observed BMD improvements are directionally consistent with pivotal clinical trial evidence and long-term extension studies showing progressive BMD gains during denosumab therapy (6–8). However, unlike highly controlled randomized trials, our study reflects routine clinical practice, including patients with previous anti-osteoporosis treatment exposure, variable adherence timing, and real-world follow-up conditions. Therefore, our results should be interpreted as complementary real-world evidence rather than as a direct comparison with randomized trial estimates.

The magnitude of BMD gain in our cohort was more modest than that reported in 10-year extension analyses (6). This difference is expected because our follow-up period was limited to 36 months and because observational cohorts may include greater clinical heterogeneity than trial populations. In response to reviewer concerns, we have avoided directly comparing 3-year outcomes with 10-year trial outcomes and instead use long-term studies only as contextual evidence.

### 4.3 Bone turnover suppression and mechanistic considerations

Denosumab directly inhibits RANKL-mediated osteoclastogenesis, leading to rapid reduction in bone resorption and subsequent improvement in BMD (6, 7). In the present study, *β*-CTX and PINP decreased substantially over 36 months, consistent with suppression of bone remodeling activity. These biomarker changes align with the known pharmacodynamic profile of denosumab and support the biological plausibility of the observed densitometric improvements (6, 8).

The increase in 25(OH)D during follow-up likely reflects calcium and vitamin D supplementation and clinical monitoring. Although serum calcium remained stable at the cohort level, mild hypocalcemia occurred in several patients. This reinforces the importance of biochemical surveillance, especially in patients with lower baseline vitamin D status, impaired renal function, or inconsistent supplementation.

### 4.4 Adherence, persistence, and delayed injections

Adherence to the six-month denosumab injection schedule is clinically important because interruption or discontinuation may cause rebound bone turnover and increase the risk of vertebral fractures (10, 11). In our cohort, all included patients completed seven denosumab injections, although delayed administration occurred in 29 patients. Most delays were within 30 days and were attributable to practical barriers such as travel, holidays, acute illness, family matters, or forgetfulness.

These findings emphasize that real-world denosumab management requires structured follow-up systems, appointment reminders, and patient education. Although no fragility fractures occurred during follow-up, our study was not designed to determine whether short injection delays affect fracture risk. Therefore, the adherence findings should be interpreted cautiously and should not be taken to imply that delayed dosing is clinically harmless.

### 4.5 Fracture outcomes and clinical implications

No incident osteoporotic fragility fractures occurred during the 36-month follow-up, whereas one traumatic tibia/fibula fracture related to an electric-bike accident was recorded and was not classified as a fragility fracture. Previous randomized studies have demonstrated fracture-risk reduction with denosumab therapy (7), but our study was not powered or designed to evaluate fracture prevention as a primary endpoint. Accordingly, the absence of incident fragility fractures should be interpreted as a descriptive safety and clinical outcome rather than definitive evidence of fracture-risk reduction.

From a clinical perspective, the combination of BMD improvement and bone turnover suppression supports continued denosumab use as part of long-term osteoporosis management. However, clinical decisions should also consider fracture history, adherence capacity, renal and calcium status, and plans for sequential therapy if denosumab is stopped (9–11).

### 4.6 Safety and monitoring

The overall safety profile was favorable. No cases of osteonecrosis of the jaw or atypical femoral fracture occurred, consistent with the low incidence of these events reported in long-term denosumab studies and reviews (8, 12). Although longitudinal mixed-effects modeling detected a statistically significant time-associated increase in serum creatinine, baseline-to-36-month paired comparisons did not reach statistical significance, and the magnitude of change remained clinically negligible. Calcium monitoring remains important during denosumab treatment, particularly in patients with low vitamin D status, impaired renal function, or inconsistent supplementation (12).

Mild hypocalcemia was observed in nine patients, and three patients developed transient hypercalcemia associated with excessive calcium or vitamin D supplementation. These calcium abnormalities were not severe and resolved after monitoring or adjustment of supplementation. These findings highlight the need for individualized supplementation guidance rather than indiscriminate high-dose calcium or vitamin D use.

### 4.7 Strengths and limitations

The strengths of this study include its longitudinal real-world follow-up, complete treatment-course data among included patients, and comprehensive assessment of skeletal outcomes, bone turnover markers, renal and mineral metabolism parameters, adherence, fracture history, and adverse events. The inclusion of adherence and safety data strengthens the real-world relevance of the findings.

Several limitations should be acknowledged. First, this was a single-center observational study without a comparator arm, limiting causal inference relative to untreated patients or alternative therapies. Second, only patients who completed seven denosumab injections and had complete evaluable follow-up data were included in the final analysis. Therefore, adherence, persistence, and safety outcomes may be overestimated compared with an intention-to-treat population. Third, 21 patients had prior anti-osteoporosis treatment exposure, which may have influenced subsequent BMD and biomarker responses despite washout periods. Fourth, fracture outcomes were not powered or adjudicated as primary endpoints, and the absence of incident fragility fractures should be interpreted cautiously. Because systematic early post-injection calcium testing was not performed for all patients, the observed hypocalcemia frequency may underestimate transient or asymptomatic calcium declines occurring shortly after denosumab administration. Finally, the results may not be fully generalizable to broader populations, men, patients with advanced chronic kidney disease, or multiethnic cohorts.

### 4.8 Future directions

Future multicenter studies should include larger sample sizes, comparator groups, intention-to-treat designs, and adjudicated fracture endpoints. Additional research is needed to determine how injection delays, prior osteoporosis treatment exposure, vitamin D status, and sequential therapy strategies influence long-term outcomes. Given the known rebound phenomenon after denosumab discontinuation, future studies should also evaluate structured transition protocols, including bisphosphonate sequencing, to reduce post-discontinuation fracture risk (10, 11).

## 5 Conclusion

In summary, 36-month denosumab therapy in postmenopausal women with osteoporosis was associated with significant BMD gains, suppression of bone turnover markers, no clinically meaningful renal deterioration, and an acceptable safety profile under routine clinical practice. These findings support denosumab as an effective intermediate-term antiresorptive therapy when accompanied by structured monitoring, adherence support, and careful planning for treatment continuation or transition. Because this analysis included only patients who completed all seven denosumab injections and had complete evaluable follow-up data, adherence, persistence, and safety outcomes should be interpreted as completer-cohort findings rather than intention-to-treat estimates.

## Data Availability

The original contributions presented in the study are included in the article/supplementary material, further inquiries can be directed to the corresponding authors.
